# Supplementary material for: Global reward state affects learning and activity in raphe nucleus and anterior insula in monkeys
Source: Nat Commun. 2020 Jul 28;11:3771. doi: 10.1038/s41467-020-17343-w (PMC7387352; doi:10.1038/s41467-020-17343-w)
Supplement: Supplementary file 1 — Supplementary Information [file 41467_2020_17343_MOESM1_ESM.pdf]

Supplementary Information

Global reward state affects learning and activity in raphe nucleus and anterior insula in monkeys

Wittmann et al.

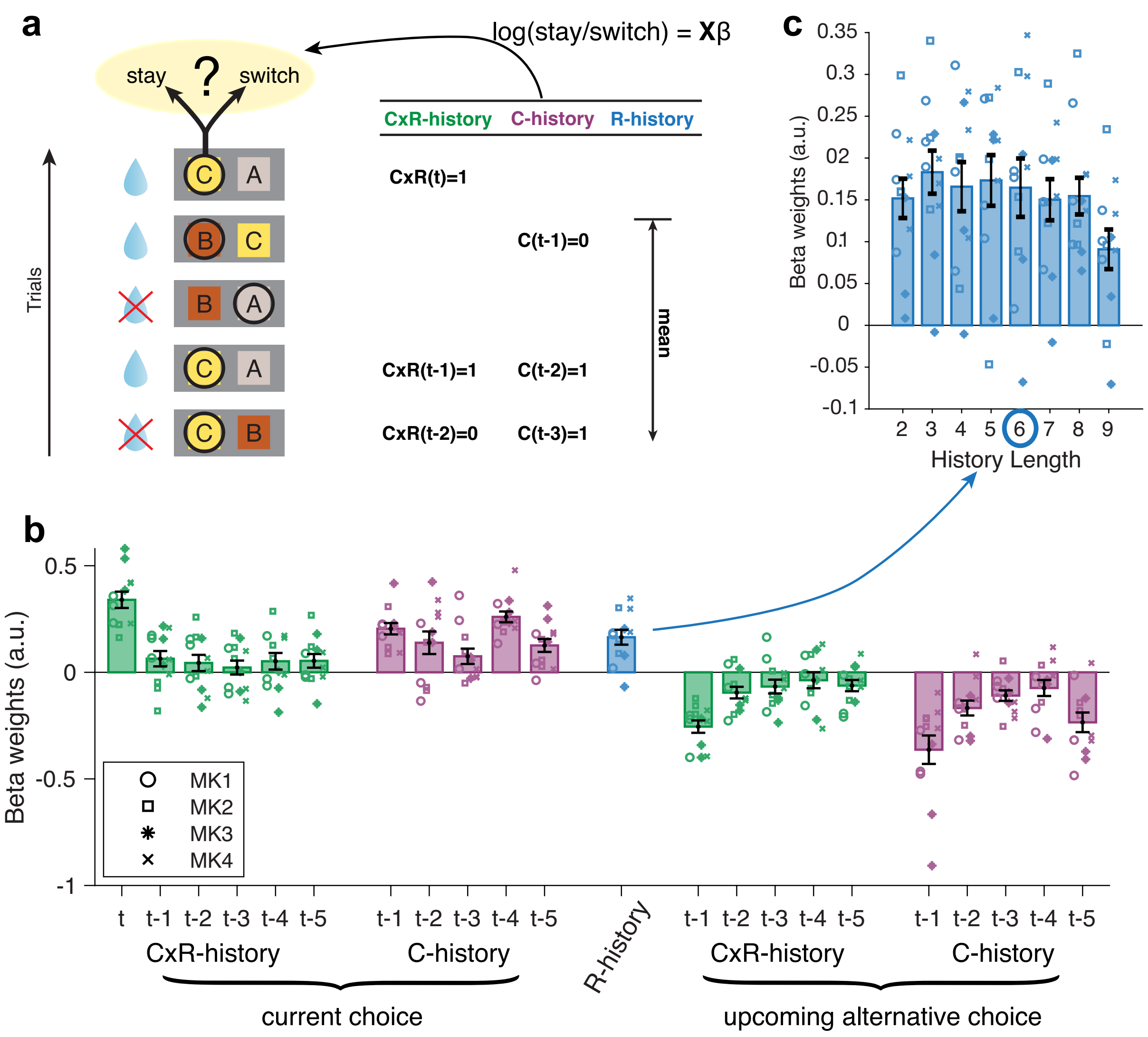

Supplementary Figure 1. Behavioral choice GLM. (**a**) Details of regressor construction relating to Figure 1e. Left: for each trial, two out of three options were offered (A, B, and C). Black circles indicate choices and (crossed through) drops on the left indicate (no) rewards. From this information, three sets of regressors were constructed. We used them to analyze for each trial t whether the animal would subsequently stay with the same choice made in this trial or switch. The three sets of regressors are illustrated on the right side: Firstly, to capture conjunctive reward effects (green, CxR-history), we entered regressors that indicated whether choices of C on trial t and also on the previous trials were rewarded or not (CxR(t), CxR(t-1), …). Note that t refers to actual occurrences of choices of C and not necessarily to consecutive trials. We only selected trials on which C was actually chosen for this regressor as only those are informative about the conjunctive choice-reward history of C. In other words, CxR-history regressors were coded for past trials in which C was chosen and set to 1/0 for rewarded/unrewarded outcomes. Reward obtained in conjunction with C should increase the probability of a stay choice. However, the stay/switch choice should also depend, inversely, on the conjunctive reward history of the alternative option that was offered as an alternative to C. To account for these effects, we created analogous regressors for the alternative option (see green weights on the right of panel b). Then, for simplicity, we aggregated the beta weights from both sets of regressors – those relating to C and those relating to the alternative option with which it was subsequently paired (see panel b for details on the aggregation). Secondly, we built regressors to account for the recent choice history of C (C(t-1), etc.). For this, we considered whether in past trials in which C was among the offered options, C was chosen or not, irrespective of any reward receipt. Again, t refers only to a subset of trials (the ones in which C was among the offered options), but a different subset compared to the CxR regressors. As the reward history for C was captured by the CxR regressors, C-history reflects the strength of the choice trace for C irrespective of whether C was recently rewarded or not. C-history regressors were coded for past trials in which C was offered and set

to 1/0 for choosing/not choosing C on that trial. Again, we constructed analogous regressors for the alternative option that would be presented with C on the next trial and aggregated the results. Finally, we took the simple average reward on the six trials before t as an index of the overall current levels of reward (R-history). This time, t refers to the actual six most recent trials as these are most relevant for estimating reward history regardless of whether C is chosen or rewarded. However, we note that the results do not change with changes in history length. (**b)** Complete set of beta weights for the GLM analysis in Fig.1f. As highlighted in panel a and in the main text, we also accounted for reward related and choice repetition related effects of the upcoming alternative option (effects on the right-hand side). For Fig.1f we sign-reversed the beta weights relating to the upcoming alternative option and averaged them with CxR-history and C-history of the current choice. We averaged the beta weights of CxR-history(t-1) of the current choice (second bar in the panel) with the beta weights from CxR-history(t-1) of the upcoming alternative (13th bar in the panel), the same for t-2 and so on. Following the same approach, we averaged the beta weights of C-history(t-1) of the current choice and the (again sign reversed) C-history(t-1) of the upcoming alternative choice, and so on for t-2, t-3, t-4 and t-5. (**c**) The effect of R-history on choice is stable across a broad window of reward history length. In our main GLM, R-history is calculated as the arithmetic mean of rewards occurring during the last six trials (see explanation in the legend of panel a). We repeated our main GLM and varied the length of this reward history between two and nine trials. The figure displays the significant effects of R-history in all of those GLMs. The blue circle indicates the R-history effect from the main GLM. The relatively weakest – but still significant – R-history effect was apparent when aggregating over nine trials ($t_{11} = 3.840$; $p = 0.003$). This might reflect the timescale over which the animals aggregate the general rewards state. However, it might also partly reflect the difficulties of estimating regression weights with a very large regressor set (the corresponding GLM contains 34 regressors). Note that varying the timescale of R-history makes it necessary to simultaneously adjust the timescale of the other relevant effects in the GLM. This will ensure that past choice-reward contingencies as well as choice history alone have a similar chance to account for the observed choices as R-history. For this reason, we concurrently varied the history length over which CxR-history and C-history were calculated. For example, when reducing the history length of R-history from six to five trials, we also reduced the history length of CxR-history and C-history by one trial (for both the current choice and the upcoming alternative choice; see description in panel a) and so on. Panels b,c concatenate sessions per monkey per experiment resulting in three data points per individual; Data are presented as mean values +/-SEM across monkey data sets; n = 12; Source data are provided as Source Data file. Symbols indicate monkey identity in panels b,c; MK abbreviates monkey.

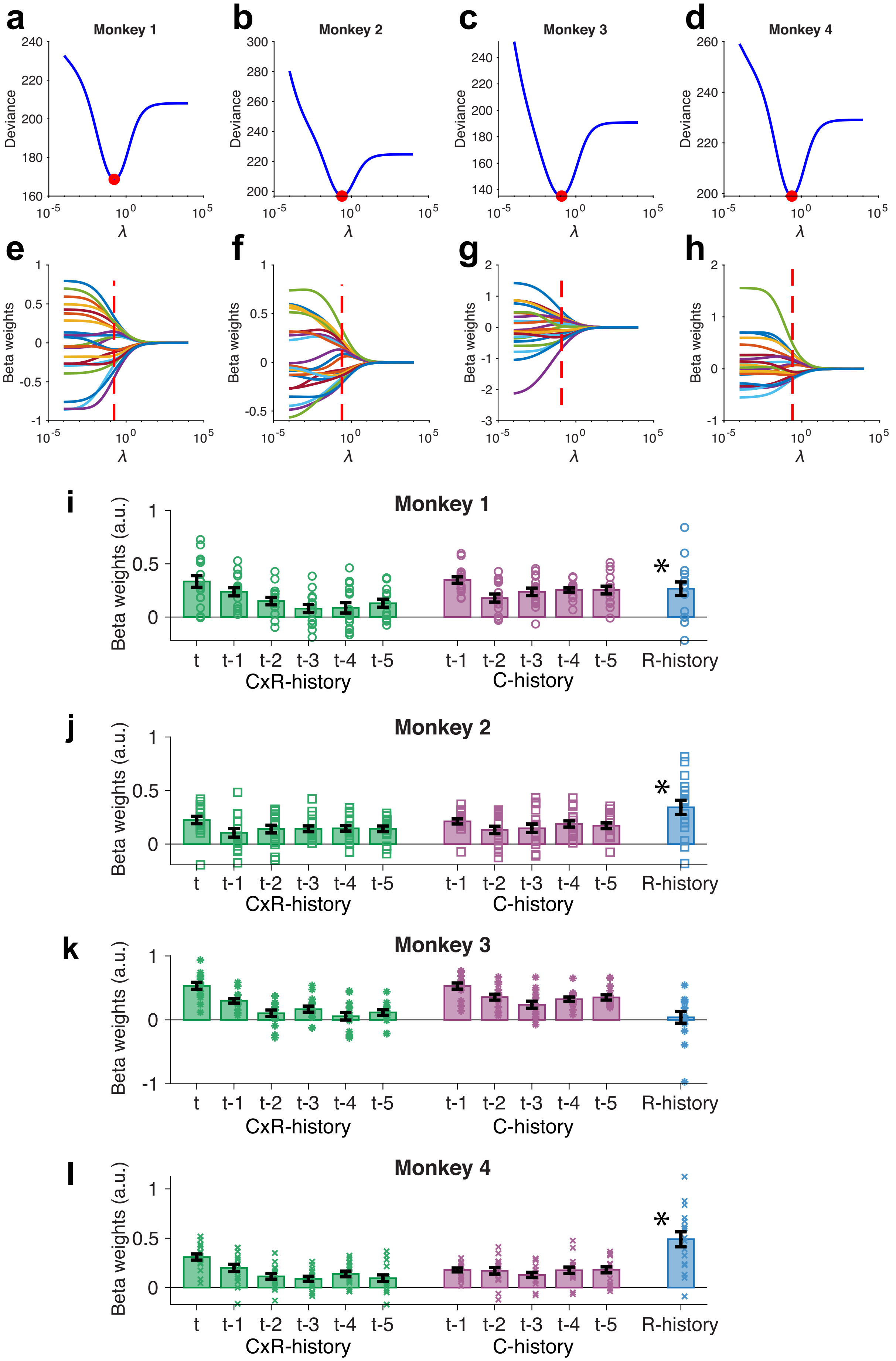
a
Monkey 1
b
Monkey 2
c
Monkey 3
d
Monkey 4
Deviance
λ
e
f
g
h
Beta weights
i
Monkey 1
j
Monkey 2
k
Monkey 3
l
Monkey 4
Beta weights (a.u.)
t
t-1
t-2
t-3
t-4
t-5
CxR-history
C-history
R-history
*

Supplementary Figure 2. Behavioral GLM analysis of individual macaques using ridge regression. To apply our regression model (shown in Fig.1f of the main manuscript) to individual macaque data, we used ridge regression[1]. Unlike in our main analysis in which we concatenated sessions, the same regression model was now applied to individual sessions. Ridge regression penalizes large beta weights according to a regularization coefficient λ and thus prevents overfitting and improves generalization. This is appropriate for cases such as ours when there are many regressors and comparatively few trials. We applied the regression model to all sessions using Matlab's lassoglm (setting Alpha to a very small value) in the following way. First, we determined an appropriate regularization coefficient λ for each individual macaque. To do this we applied a five-fold cross-validation to the data sampling over a large space of λ from zero to 10^{-4} to 10^{4} (log spaced) to each individual session. We repeated this procedure 10 times and determined the overall model deviance for each λ for all sessions from the same monkey (irrespective of experiment) combined. We then selected the λ for each individual macaque that resulted in the lowest overall model deviance for that monkey; this is the λ for the best cross-validated model fit. A single λ was used for all sessions from the same monkey to keep regression weights to scale. **(a-d)** Deviance averaged across sessions plotted over λ in log space. The red dot indicates the point of lowest deviance (i.e. best cross-validated model fit), which was used to pick the monkey specific λ. The resulting λs for the four monkeys were close together: 0.17074 (monkey 1), 0.24771 (monkey 2) 0.11768 (monkey 3), 0.24771 (monkey 4) and reflect the best trade-off of model parsimony and predictive power. **(e-h)** All beta weights (except the model intercept) averaged over sessions and plotted over log spaced λ. The vertical red line indicates the λ used for the monkey. Note that beta weights decrease along the λ axis as the penalty for large beta weights increases. Panels in the same column correspond to the same individual **(i-l)** After selection of macaque-specific λs in such a way, we applied the regression model using these λs. Then, again, we tested our critical effect of interest, the R-history effect, in all monkeys to determine whether we were able to find evidence for it on the individual monkey level. Indeed, we found a significant effect of R-history in three out of four monkeys using two-sided one sample t-tests against zero. (monkey 1, n=16: $t_{15} = 4.210$; $p < 0.001$; monkey 2, n=17: $t_{16} = 5.201$; $p < 0.001$; monkey 3, n=15: $t_{14} = 0.425$; $p = 0.677$; monkey 4,n=17: $t_{16} = 6.426$; $p < 0.001$). Data are presented as mean values +/-SEM; asterisks indicate $p< 0.05$). Source data are provided as Source Data file. Symbols in panels I,j,k,l indicate monkey identity according to the same convention as in all other figures.

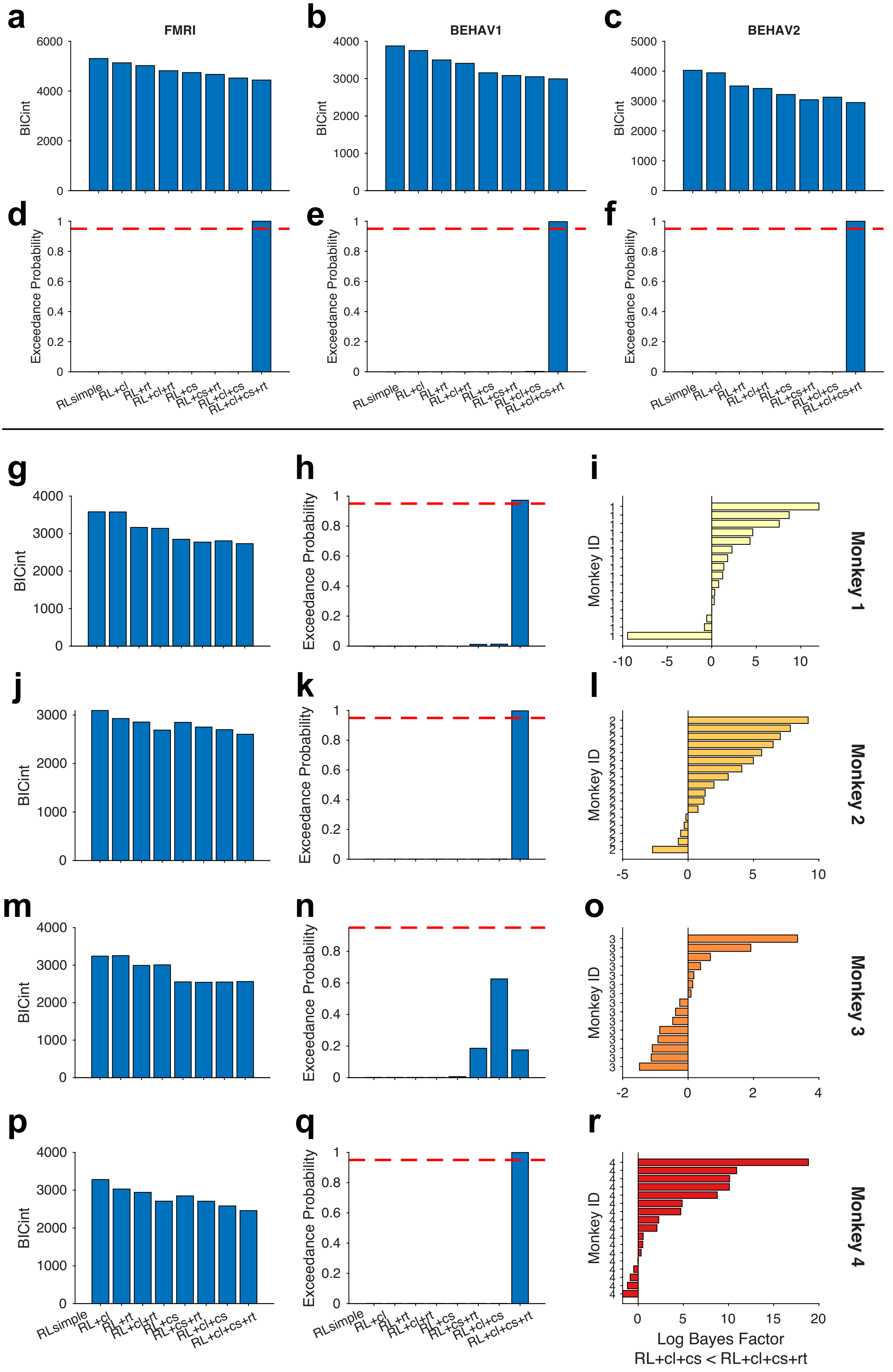
a
FMRI
BICint
b
BEHAV1
c
BEHAV2
d
Exceedance Probability
e
f
RLsimple
RL+cl
RL+rt
RL+cl+rt
RL+cs
RL+cs+rt
RL+cl+cs
RL+cl+cs+rt
g
h
i
Monkey ID
Monkey 1
j
k
l
Monkey 2
m
n
o
Monkey 3
p
q
r
Monkey 4
Log Bayes Factor
RL+cl+cs < RL+cl+cs+rt

Supplementary Figure 3. Supplementary model comparison over experiments and monkeys. For these analyses, the sessions were resorted according to the experiment they were recorded in irrespective of monkey identity (a-f) or according to the identity of monkey irrespective of experiment (g-r). **(a-f)** Supplementary model comparison for individually fitted experiments. BICint and exceedance probability (XP) for the fMRI data set (**a,d**) and the two behavioral data sets (**b,e** and **c,f**, respectively). According to both indices, the full model best explained the observed choice patterns among the set of tested models. The XPs of the full model are: XP-FMRI = 0.990; XP-BEHAV = 0.997; XP-BEHAV2 = 0.999. Note that the log Bayes factors for the best-fitting compared to the second-best model for each experiment are displayed in the main manuscript. (n=25 sessions for the fMRI experiments in a and d; and n=20 for both behavioural experiments shown in the remaining panels) **(g-r)** Supplementary model comparison for individually fitted monkeys. BICint, XPs, and log Bayes factor of the overall best-fitting compared to the second-best fitting model are shown in separate columns for monkey 1 (g-i), monkey 2 (j-l), monkey 3 (m-o) and monkey 4 (p-r). The x-labels are the same for all panels of the same column and are displayed in the last row. For monkeys 1,2, and 4, both the BICint and the XP clearly favour the full model. The XPs of the full model are: XP-monkey1 = 0.973, XP-monkey2 = 0.998, XP-monkey4 = 0.999. By contrast, for monkey 3, there are divergent results when considering the XP or the BICint. Regarding the XP, no model reaches an XP higher than 0.95; the relative best model for monkey 3 is one that does not contain a reward trace (XP-monkey3-RL+cl+cs=0.625), followed by two models that contain a reward trace (RL+cs+rt and RL+cl+cs+rt). By contrast, according to the BICint, the marginally best model is one that contains a reward trace (BICint-monkey3-RL+cs+rt= 2543.8) followed by one that does not contain one (BICint-monkey3-RL+cl+cs=2549.4). Note that monkey 3 is the same animal for which there was no clear effect of the global reward state in the corresponding behavioral analyses (R-history effect in Supplementary Fig.2k), while the remaining three animals clearly showed such an effect. In conclusion, there is no clear winning model when considering both the BICint and the XP together for monkey 3. However, it suggests that monkey 3's choices were less influenced by the global reward state compared to the other monkeys. All together and in accordance with the behavioral analyses of individual monkeys, these results clearly demonstrate that the global reward state effects described in this manuscript are not driven by single outlying individuals. Note that the precise BICint and XP values for all panels are provided in the source data file related to this manuscript. The dashed red line in panels displaying exceedance probabilities indicates an exceedance probability of 0.95. Source data are provided as Source Data file. (n=16 sessions for monkey1 in panels g,h,i; n=17 sessions for monkey 2 in panels j,k,l; n=15 sessions for monkey 3 in panels m,n,o; n=17 sessions for monkey 4 in panels p,q,r).

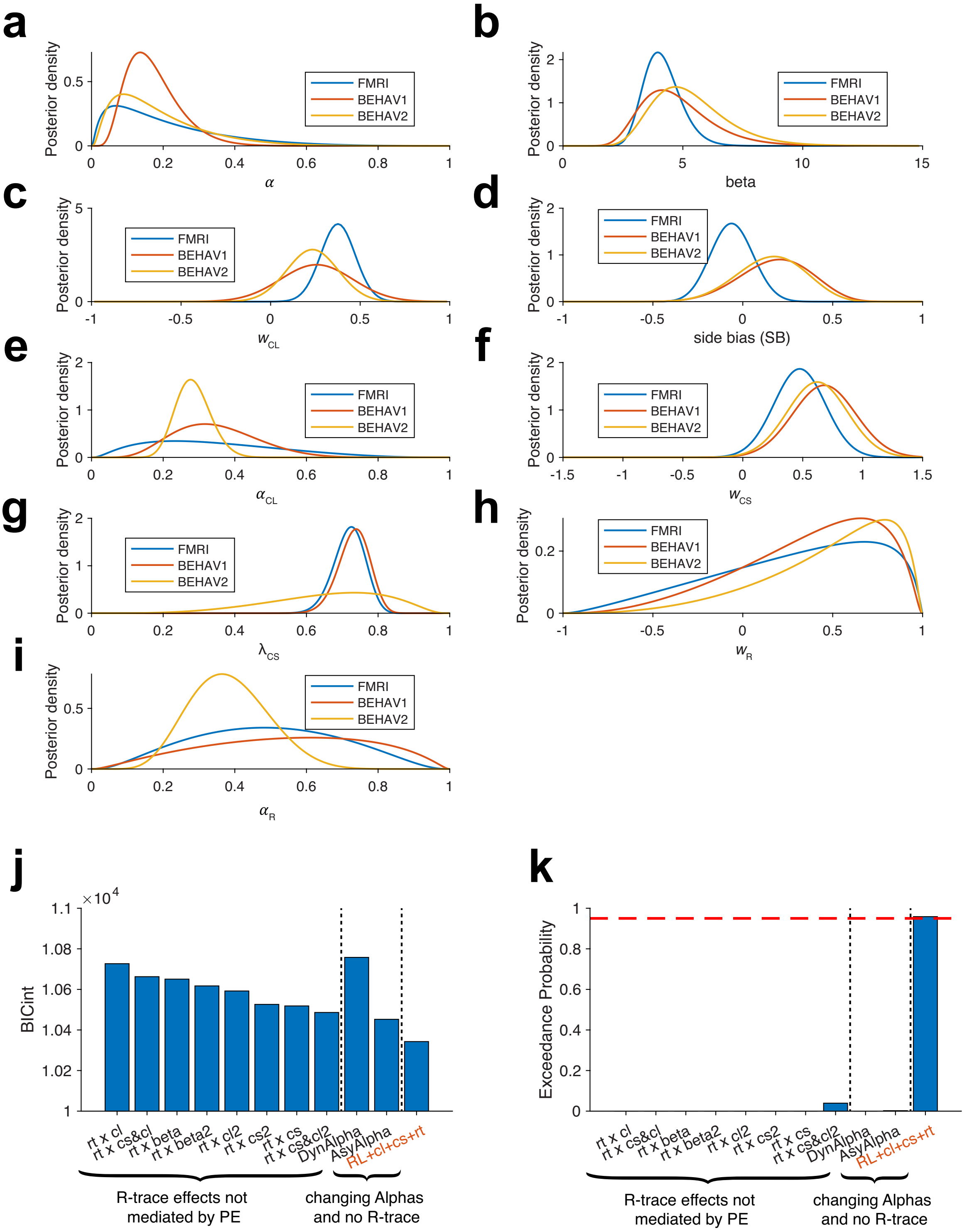

Supplementary Figure 4. Supplementary modelling information. (**a-i**) Parameter distributions for all free parameters of the full model RL+cl+cs+rt. The model was fitted individually for each experimental data set. See main text for details on panel h showing the weight of R-trace in the prediction error calculation. (**j,k**) Supplementary control model analyses showing BICint and exceedance probabilities for the full model as well as all supplementary models. The model comparison includes all supplementary models as explained in the Methods section. There are two broad categories of control models: models that assume an influence of R-trace, but one that is not mediated by PEs but by different mechanisms; and a second category that assumes no R-trace, but instead that value updates have different dynamics. The full model RL+cl+cs+rt is the winning model according to both BICint and exceedance probability (exceedance probability = 0.959). The red line indicates an exceedance probability of 0.95. Source data are provided as Source Data file. (n = 65 sessions)

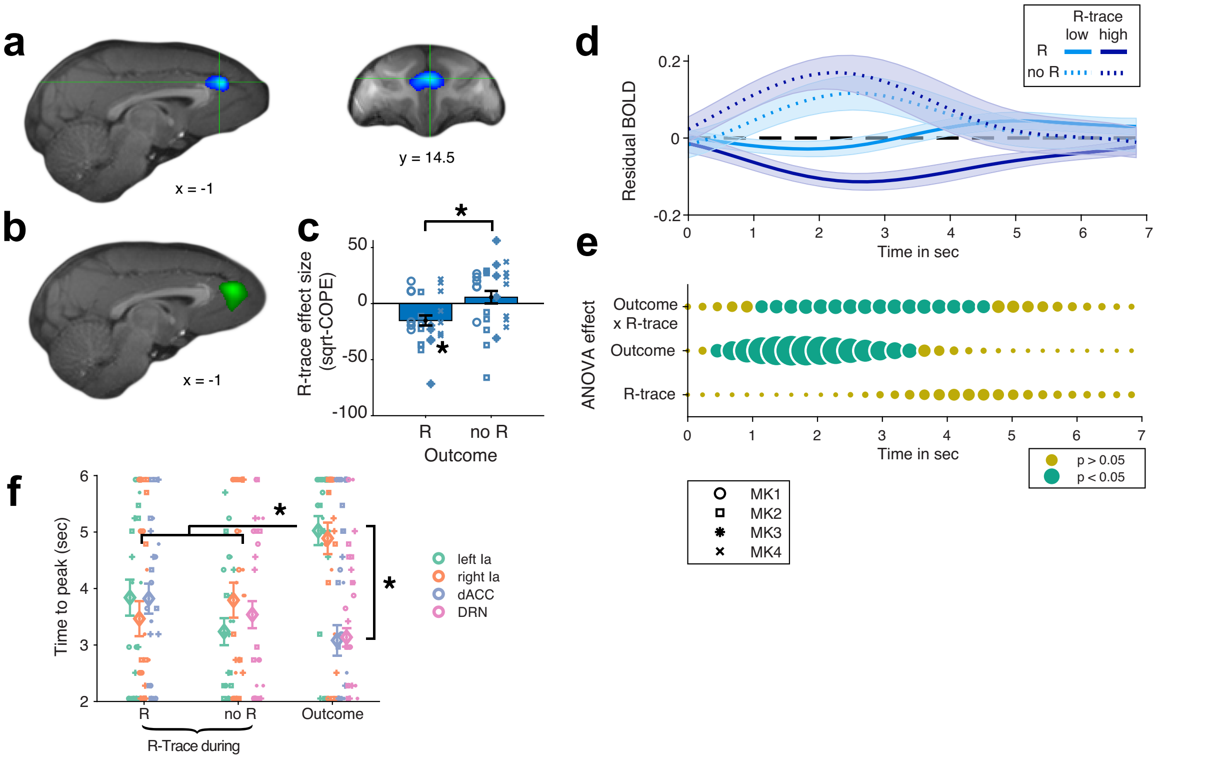

Supplementary Figure 5. Supplementary neural analyses of R-trace and outcomes. (**a**) R-trace, calculated over all trials (GLM1), identified a significant negative cluster of activation in dorsal anterior cingulate cortex (dACC). (**b**) Such processing of recent rewards was complemented with (negative) signals relating to current outcome that were correlated with dACC activity in a more anterior portion of the cingulate sulcus. Note that R-trace signals and outcome signals in dACC were identified via the same GLM. (**c**) ROI analyses in dACC (see crosshair in panel a) revealed that R-trace signals in dACC were significant only during rewarded (t_{24} =3.32; p = 0.003; two-sided one sample t-test against zero), but not during unrewarded trials (t_{24} =1.00; p = 0.33; two-sided one sample t-test against zero), with a significant difference between them (t_{24} =2.96; p = 0.007; two-sided paired t-test). Note that we used a leave-one-out procedure to identify session-specific ROIs. This pattern of effects is opposite to the pattern observed in DRN. (**d,e**) Time course analyses of dACC activity. Same conventions as in figures 7 and 8. (**f**) Time to peak for R-trace and outcome ANOVA effects for bilateral Ia, DRN and dACC. One intriguing difference in coding schemes between, on the one hand, Ia and, on the other hand, DRN and dACC was the late timing of outcome encoding in Ia. Rather than exerting an effect after 3-4 seconds, i.e. the approximate peak of the macaque hemodynamic response function (HRF), outcome signals emerged after 5 seconds in both left and right Ia. On average, Ia outcome signals emerged significantly later than the average R-trace peak in Ia (t_{24} =4.26; $p < 0.001$; two-sided paired t-test), or the R-trace signals in DRN (in unrewarded trials: t_{24} =4.82; $p < 0.001$; two-sided paired t-test) and dACC (in reward trials t_{24} =3.07; p = 0.005; two-sided paired t-test), all of which emerged close in time to the expected HRF peak. Strikingly, such late processing of reward outcomes in Ia also differed from much earlier outcome signals in DRN (t_{24} =7.25; $p <$ 0.001; two-sided paired t-test) and dACC (t_{24} =5.89; $p < 0.001$; two-sided paired t-test). This might suggest that outcomes in Ia are represented at a time at which they no longer define the current state of the animal but have themselves become part of the GRS. Note that dACC and DRN R-trace effects are only shown for rewarded and unrewarded trials, respectively, because R-trace effects were only observed under these conditions in these ROIs. (Crosshairs highlight peak coordinates for subsequent ROI analysis; Data are presented as mean values +/-SEM across sessions; n=25; dot color in c indicate monkey identity; all MRI results cluster-corrected at Z>2.6, p=0.05; asterisks indicate p < 0.05). Source data are provided as Source Data file. Symbols indicate monkey identity in panels c,f; MK abbreviates monkey.

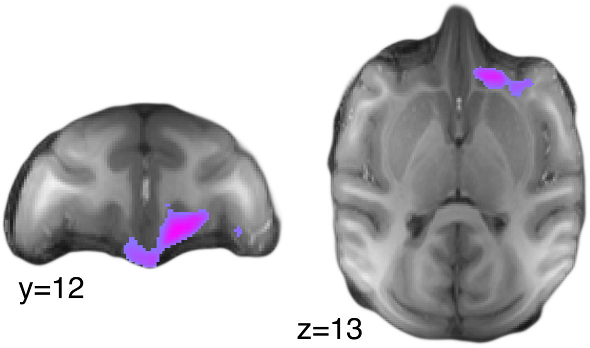

Supplementary Figure 6. Choice trace signals in medial orbitofrontal cortex. We attempted to specify the type of choice history representation present in prefrontal cortex more precisely. As shown in the behavioral analyses and modelling, the monkeys in our experiments had a strong tendency, irrespective of reward, to stay with previously picked stimuli. The experimental design allows dissociation of this tendency, indexed by the CS-trace variable from the tendency to just repeat choice location. We regressed the BOLD activity against the relative strength of the chosen choice stimulus trace (CS-trace(chosen-unchosen); GLM1). CS-trace(chosen-unchosen) captures the degree to which the animals stay with their previous choice stimulus or switch away to a stimulus they have not picked for a while. We found a cluster of activity overlapping but slightly adjacent to previous vmPFC/mOFC activity in a posterior part of medial orbitofrontal cortex (mOFC). Again, chosen and unchosen option effects manifested with the same negative and positive signs as observed for chosen and unchosen value effects in vmPFC/mOFC. (n=25 sessions; MRI results cluster-corrected at Z>2.6, p=0.05).

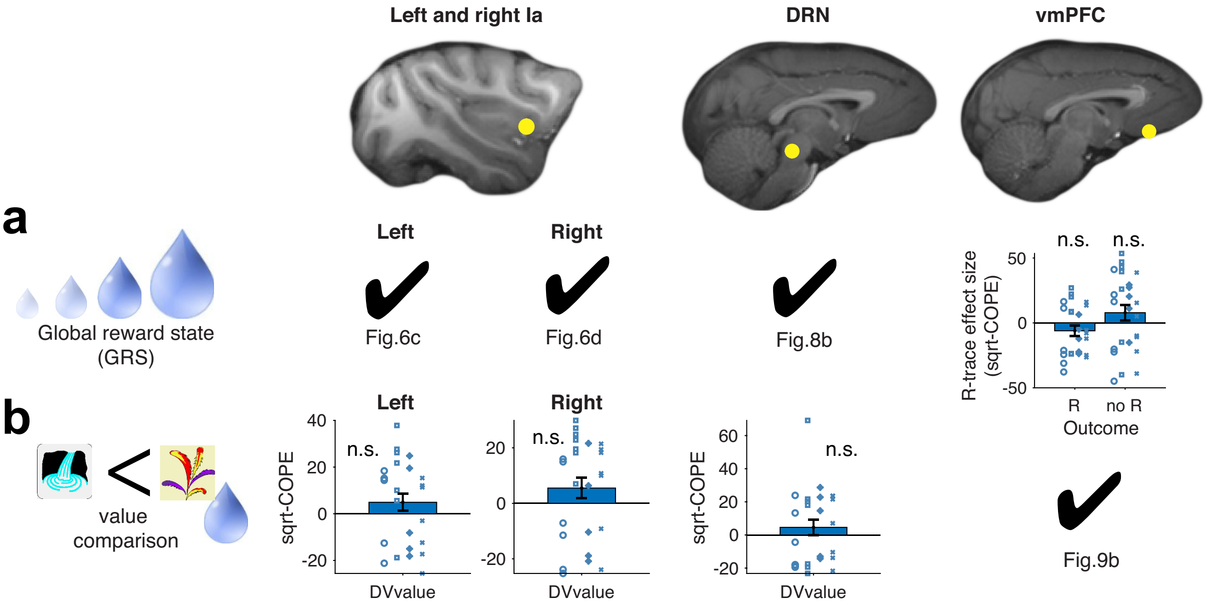

Supplementary Figure 7. Summary analysis of sqrt-COPE images in previously established ROIs. (**a**) Examining R-trace, we have found evidence for a global reward state representation in Ia bilaterally and in DRN (see references to main figures below the check marks). In vmPFC, however, we did not find a R-trace signal, neither during rewarded (t_{24} =-1.56; p = 0.132) nor unrewarded trials (t_{24} =-1.56; p = 0.132). We used the vmPFC ROI identified by DV_{total}. We did not use a leave-one-out-procedure to identify the ROI since the tested contrast was independent of the ROI defining contrast. (**b**) In contrast, representations of the value comparison, the chosen minus unchosen value or DV_{value}, were very clear in vmPFC (see reference to main text figure). However, we did not find such signals in the left Ia (t_{24} =-1.34; p = 0.193), right Ia (t_{24} =1.50; p = 0.148), or DRN (t_{24} =0.99; p = 0.331). Ia and DRN ROIs were identified by the main effect of R-trace for Ia and by the effect of R-trace during unrewarded trials in DRN; again, not using a leave-one-out procedure since we were examining effects that were independent of ROI selection. (Data are presented as mean values +/-SEM across sessions; n=25; dot color in c indicate monkey identity). Source data are provided as Source Data file. Symbols indicate monkey identity; MK abbreviates monkey.

Contrast	Region	Peak Coordinates x/y/z (in mm)	Z-Value
R-trace (positive contrast; GLM1)	**Right agranular insula**	19.5/4.5/-5	4.58
	**Left agranular insula**	-18/3.5/-1	4.43
	Cerebellum	14/-35/-20.5	4.14
R-trace (negative contrast; GLM1)	**Dorsal anterior cingulate cortex (dACC)**	-1/14.5/13.5	3.94
	Right dorsolateral prefrontal cortex	14/6.5/11.5	3.85
	Right posterior superior temporal sulcus / posterior parietal cortex	18.5/-33.5/13.5	3.85
	Left posterior superior temporal sulcus/ posterior parietal cortex	-24/-25/2	4.04
Outcome (positive contrast; Reward < no Reward; GLM1)	Dorsal anterior cingulate cortex (dACC)	0/19/11	4.31
Outcome (positive contrast; Reward > no Reward; GLM1)	Left ventral premotor cortex	-22/-6/8	6.48
	Right ventral premotor cortex	23/-5.5/8.5	5.65
	Right Amygdala	11.5/-5/-11	6.1
	Left Amygdala	-11.5/-5.5/-6.5	5.37
	Left Putamen	-13.5/0/-4	5.3
	Right Putamen	14/-2/-2.5	4.71
	Left insula	-22/0.5/2	4.94
	Right insula	18.5/1.5/-1	3.95
	Medial dorsal Thalamus	2/-9/2	4.61
	Ventral tegmental area	1/-8.5/-8.5	4.57
	Pons	1.5/-14/-14.5	4.65
R-trace during unrewarded trials (negative contrast; GLM 2)	Left Cerebellum	-11.5/-25.5/-11	3.94
	**Dorsal nucleus raphe (DRN)**	1/-16/-8	3.49
CS-trace (chosen-unchosen) (negative contrast; GLM1)	Left medial orbitofrontal cortex (mOFC)	-6/11.5/1.5	3.93

DV_{total} (negative contrast; GLM 3)	Right lateral orbitofrontal cortex	14.5/16.5/9.5	4.98
	Perigenual anterior cingulate cortex	15.5/20/10	4.10
	**Ventromedial prefrontal cortex (vmPFC)**	-1/12.5/-3	3.80
DV_{value} (negative contrast; GLM4)	ventromedial prefrontal cortex (vmPFC)	1.5/13.5/-2	3.39
	Left orbitofrontal cortex	-13/19.5/9.5	3.58
	visual Cortex	2.5/-37/-0.5	4.17
	Lunate Sulcus	-22.5/-32.5/7	3.87

Supplementary Table 1. Peak coordinates of activation clusters. Coordinates are given according to the CARET macaque F99. Regions of interest analyses were performed in regions highlighted in bold.

References:

1. Huth, A. G., de Heer, W. A., Griffiths, T. L., Theunissen, F. E. & Gallant, J. L. Natural speech reveals the semantic maps that tile human cerebral cortex. *Nature* **532**, 453–8 (2016).
