# Supplementary material for: Global reward state affects learning and activity in raphe nucleus and anterior insula in monkeys
Source: Nat Commun. 2020 Jul 28;11:3771. doi: 10.1038/s41467-020-17343-w (PMC7387352; doi:10.1038/s41467-020-17343-w)
Supplement: Supplementary file 3 — Reporting Summary [file 41467_2020_17343_MOESM3_ESM.pdf]

nature research

Corresponding author(s): Marco Wittmann

Last updated by author(s): May 15, 2020

Reporting Summary

Nature Research wishes to improve the reproducibility of the work that we publish. This form provides structure for consistency and transparency in reporting. For further information on Nature Research policies, seeAuthors & Referees and theEditorial Policy Checklist .

Statistics

For all statistical analyses, confirm that the following items are present in the figure legend, table legend, main text, or Methods section.

n/a	Confirmed	
☐	☒	The exact sample size (*n*) for each experimental group/condition, given as a discrete number and unit of measurement
☐	☒	A statement on whether measurements were taken from distinct samples or whether the same sample was measured repeatedly
☐	☒	The statistical test(s) used AND whether they are one- or two-sided<br>*Only common tests should be described solely by name; describe more complex techniques in the Methods section.*
☐	☒	A description of all covariates tested
☐	☒	A description of any assumptions or corrections, such as tests of normality and adjustment for multiple comparisons
☐	☒	A full description of the statistical parameters including central tendency (e.g. means) or other basic estimates (e.g. regression coefficient) AND variation (e.g. standard deviation) or associated estimates of uncertainty (e.g. confidence intervals)
☐	☒	For null hypothesis testing, the test statistic (e.g. *F*, *t*, *r*) with confidence intervals, effect sizes, degrees of freedom and *P* value noted<br>*Give P values as exact values whenever suitable.*
☐	☒	For Bayesian analysis, information on the choice of priors and Markov chain Monte Carlo settings
☐	☒	For hierarchical and complex designs, identification of the appropriate level for tests and full reporting of outcomes
☐	☒	Estimates of effect sizes (e.g. Cohen's *d*, Pearson's *r*), indicating how they were calculated

Our web collection on statistics for biologists contains articles on many of the points above.

Software and code

Policy information about availability of computer code

Data collection	Stimuli were presented and choices recorded via Presentation software (Neurobehavioral Systems Inc., Albany, CA; version 16). Functional Resonance Magnetic Imaging (fMRI) data were acquired using a 3T MRI scanner and a four-channel phased array receive coil in conjunction with a radial transmission coil (Windmiller Kolster Scientific Fresno, CA). Data is available at: https://osf.io/358cg/?view_only=0e6fda7925364d86930374cd4ae4a59f
Data analysis	We analyzed data using Matlab 2018a version 9.4.0, Jasp version 0.9.0.1 and FSL version 5.0.011. For Bayesian comparison we used the function spm_BMS (Statistical Parametric Mapping 12).

For manuscripts utilizing custom algorithms or software that are central to the research but not yet described in published literature, software must be made available to editors/reviewers. We strongly encourage code deposition in a community repository (e.g. GitHub). See the Nature Research guidelines for submitting code & software for further information.

Data

Policy information about availability of data

All manuscripts must include a data availability statement. This statement should provide the following information, where applicable:
- Accession codes, unique identifiers, or web links for publicly available datasets
- A list of figures that have associated raw data
- A description of any restrictions on data availability

We have deposited all behavioral raw data used for the reinforcement learning model in an OSF repository. The repository also comprises the full reinforcement modelling pipeline including model comparisons. All model-derived variables that are used for the MRI analyses are derived from this pipeline. In addition, Matlab code to repeat the basic behavioural GLM is provided. Accession code to the repository is the following and a README inside the repository explains the details of its use:
https://osf.io/358cg/?view_only=0e6fda7925364d86930374cd4ae4a59f
WWe have also deposited all group-level contrast images presented in the manuscript on Neurovault. The accession code is: https://neurovault.org/collections/

KJVDIJYY/. Any remaining data that support the findings of this study are available from the corresponding author upon reasonable request

Field-specific reporting

Please select the one below that is the best fit for your research. If you are not sure, read the appropriate sections before making your selection.

- [x] Life sciences
- [ ] Behavioural & social sciences
- [ ] Ecological, evolutionary & environmental sciences

For a reference copy of the document with all sections, see nature.com/documents/nr-reporting-summary-flat.pdf

Life sciences study design

All studies must disclose on these points even when the disclosure is negative.

Sample size	25 sessions were performed in an MRI scanner. Each animal performed between 5 to 7 fMRI scans. No statistical methods were used to pre-determine sample sizes but our sample size for the MRI analyses are in accordance with gold standards as described in (Friston, Neuroimage, 1999; Desmond, Journal of Neuroscience Methods, 2002) and our previous work (Chau, B. K. H. et al. Neuron, 2015; Papageorgiou, G. K. et al. Nat. Commun., 2017).
Data exclusions	No session were excluded from the analysis.
Replication	For behavioral analyses and modelling, we pool data from three experiments, two behavioral and one fMRI experiment comprising overall 65 sessions of task-related choice data from four healthy male rhesus monkeys (Macaca mulatta). Key behavioral analyses replicated over all three experiments.
Randomization	We used a within subject design and no randomization was performed.
Blinding	We used a within subject design and therefore blinding was not relevant.

Reporting for specific materials, systems and methods

We require information from authors about some types of materials, experimental systems and methods used in many studies. Here, indicate whether each material, system or method listed is relevant to your study. If you are not sure if a list item applies to your research, read the appropriate section before selecting a response.

Materials & experimental systems

n/a	Involved in the study	
x		Antibodies
x		Eukaryotic cell lines
x		Palaeontology
	x	Animals and other organisms
x		Human research participants
x		Clinical data

Methods

n/a	Involved in the study	
x		ChIP-seq
x		Flow cytometry
	x	MRI-based neuroimaging

Animals and other organisms

Policy information about studies involving animals; ARRIVE guidelines recommended for reporting animal research

Laboratory animals	Four male rhesus monkeys (Macaca mulatta) were involved in the experiment. They weighed 10.4–11.9 kg and were 7 years of age. They were group housed and kept on a 12 hr light dark cycle, with access to water 12–16 hr on testing days and with free water access on non-testing days.
Wild animals	The study did not involve wild animals.
Field-collected samples	The study did not involve samples collected from the field.
Ethics oversight	All procedures were conducted under licenses from the United Kingdom (UK) Home Office in accordance with the UK The Animals (Scientific Procedures) Act 1986 and with the European Union guidelines (EU Directive 2010/63/EU).

Note that full information on the approval of the study protocol must also be provided in the manuscript.

Magnetic resonance imaging

Experimental design

Design type	Event related fMRI design
Design specifications	Animals had to choose repeatedly between different stimuli that were novel in each testing session (Figure 1). Each session comprised 200 trials. We used a probabilistic reward-based learning task. The task consisted of a series of choices, on each trial, between two stimuli drawn out of a larger pool of three. The position of the two available options on the left and right side of the screen were fully randomized. Animals had to choose any symbols by touching one of two infra-red sensors placed in front of their two hands corresponding to the stimuli on the screen. After making their decision, if the correct option was selected, the unselected option disappeared and the chosen option remained on the screen and a juice reward was delivered. If an incorrect choice was made, no juice was delivered. The outcome phase lasted 1.5 seconds. Each reward was composed of two 0.6 ml drops of blackcurrant juice delivered by a spout placed near the animal's mouth during scanning. The experiment was controlled by Presentation software (Neurobehavioral Systems Inc., Albany, CA).
Behavioral performance measures	200 responses were recorded in each session as well as reaction times. We fitted a reinforcement learning model to the animal choices. The fitting procedure is described in the Methods. We also used multiple logistic and linear regressions to explain behavioral responses.

Acquisition

Imaging type(s)	Functional and structural
Field strength	3T
Sequence & imaging parameters	Awake-animals were head-fixed in a sphinx position in an MRI-compatible chair. We collected fMRI using a 3T MRI scanner and a four-channel phased array receive coil in conjunction with a radial transmission coil (Windmiller Kolster Scientific Fresno, CA). FMRI data were acquired using a gradient-echo T2* echo planar imaging (EPI) sequence with 1.5 x 1.5 x 1.5 mm3 resolution, repetition time (TR) = 2.28 s, Echo Time (TE) = 30 ms, flip angle = 90, and reference images for artifact corrections were also collected. Proton-density-weighted images using a gradient-refocused echo (GRE) sequence (TR = 10 ms, TE = 2.52 ms, flip angle = 25) were acquired as reference for body motion artifact correction. T1-weighted MP-RAGE images (0.5 x 0.5 x 0.5 mm3 resolution, TR = 2,5 ms, TE = 4.01 ms) were acquired in separate anesthetized scanning sessions.
Area of acquisition	whole-brain

Diffusion MRI ☐ Used ☒ Not used

Preprocessing

Preprocessing software	The preprocessing of the fMRI data used tools of FSL (https://fsl.fmrib.ox.ac.uk/fsl/fslwiki) and the Magnetic Resonance Comparative Anatomy Toolbox (MrCat; http://www.rbmars.dds.nl/lab/toolbox.html).
Normalization	Linear and non-linear registration to F99 space was achieved using FLIRT (Jenkinson et al., 2002; Jenkinson and Smith, 2001) and FNIRT (Andersson et al., 2007; Jenkinson et al., 2012) with configurations adjusted to reflect macaque rather than human brain characteristics.
Normalization template	We used the macaca mulatta F99 template in Caret (Van Essen, 2002; Van Essen and Dierker, 2007) as our group template.
Noise and artifact removal	FMRI data were corrected for body motion artefacts by an offline-SENSE reconstruction method 5 (Offline_SENSE GUI, Windmiller Kolster Scientific, Fresno, CA). The images were aligned to an EPI reference image slice-by-slice to account for body motion and then aligned to each animal's structural volume to account for static field distortion 6 (Align_EPI GUI and Align_Anatomy GUI, Windmiller Kolster Scientific, Fresno, CA). The aligned data were processed with high-pass temporal filtering (3-dB cutoff of 100s) and Gaussian spatial smoothing (full-width half maximum of 3mm). The data that were already registered to each subject's structural space were then registered to the CARET macaque F99 template7 using affine transformation.
Volume censoring	We did not remove volumes during which significant movement occurred, instead, we used our motion-related artifacts (i.e. regression of motion parameters) as regressors of non interest that were not convolved in our general linear models.

Statistical modeling & inference

Model type and settings	We employed a univariate approach within the general linear model framework to perform whole-brain statistical analyses of functional data as implemented in the FMRIB Software Library. Using this framework we initially performed a first-level fixed effects analysis to process each individual experimental run which were then combined in a second level mixed-effects analysis treating sessions as a random effects.

Effect(s) tested

The full models are described in the methods including the nature of all regressors entered in the analyses. See GLM1, GLM2, GLM3 and GLM4.

Specify type of analysis: ☐ Whole brain ☐ ROI-based ☒ Both

Anatomical location(s)

Anatomical locations were determined via peak coordinates of whole-brain significant clusters and identified using "The rhesus monkey brain in stereotaxix coordinates" (Paxinos, Huang & Toga, 200).

Statistic type for inference
(See Eklund et al. 2016)

We performed a cluster inference using a cluster-defining threshold with |Z| > 2.6.

Correction

Family-wise error cluster correction (z > 2.6 and p = 0.05).

Models & analysis

n/a	Involved in the study
☒	☐ Functional and/or effective connectivity
☒	☐ Graph analysis
☒	☐ Multivariate modeling or predictive analysis
